# Effects of Electric Potential Treatment of a Chromium Hexacyanoferrate Modified Biosensor Based on PQQ-Dependent Glucose Dehydrogenase

**DOI:** 10.3390/s100706347

**Published:** 2010-06-28

**Authors:** Ta-Feng Tseng, Yang-Li Yang, Yuh-Jiuan Lin, Shyh-Liang Lou

**Affiliations:** 1 Biomedical Engineering Department, Chung-Yuan Christian University, No. 200, Zhongbei Rd., Zhongli City, Taoyuan County 32023, Taiwan; E-Mails: g9402502@cycu.edu.tw (T.F.T); g9275023@cycu.edu.tw (Y.L.Y); 2 Medical Electronics and Device Technology Center, Industrial Technology Research Institute, Rm. 334, Bldg. 53, 195, Sec. 4, Chung Hsing Rd., Chutung, Hsinchu County 31040, Taiwan; E-Mail: yjlin@itri.org.tw

**Keywords:** biosensor, chromium hexacyanoferrate, PQQ-dependent glucose dehydrogenase, mediator, potential treatment

## Abstract

A novel potential treatment technique applied to a glucose biosensor that is based on pyrroloquinoline quinone (PQQ)-dependent glucose dehydrogenase (GDH) and chromium hexacyanoferrate (CrHCF) incorporated into a platinum (Pt) electrode was demonstrated. CrHCF, serving as a mediator, was electrochemically deposited on the Pt electrode as ascertained by CV, SEM, FTIR and XPS measurements. The potential treatment of CrHCF, which converts Fe(II) to Fe(III), enables the glucose detection. The amperometric measurement linearity of the biosensor was up to 20 mM (*R* = 0.9923), and the detection sensitivity was 199.94 nA/mM per cm^2^. More importantly, this biosensor remained stable for >270 days.

## 1. Introduction

The reaction of the pyrroloquinoline quinone (PQQ)-dependent glucose dehydrogenase (PQQ-GDH) enzyme system with glucose requires no oxygen, which in turn means that no hydrogen peroxide is produced [1–3]. These features make this enzyme system attractive in applications where oxygen supply is limited. The active site of the PQQ-GDH enzyme system is deep within its three-dimensional structure, causing slow electron transfer from PQQ, which is adjacent to the active site of GDH, to an electrode surface when the glucose is oxidized by the enzyme. This slow transfer typically results in small signal responses. Hence, the development of PQQ-GDH based glucose biosensors with suitable redox mediators has drawn the attention of many research groups.

Mediators that have been adopted in the implementation of PQQ-GDH biosensors with useful response signals include osmium complexes [4–6], ruthenium complexes [7,8], *N*-methyl-phenazonium [9], ferrocene derivatives [10], cytochrome b562 [11], ferricyanide [12], carbon nanotubes [13] and gold nanoparticles [14]. Polymers such as polypyrrole [15–17], polyurethane [18] and poly(acrylate) [19] have been used for the immobilization of PQQ-GDH enzymes.

**Scheme 1.**
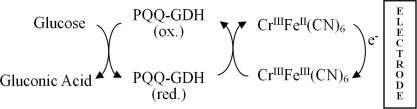
Proposed glucose detection mechanism.

To intensify the signals, most of these biosensors use electrodes with large surface areas, such as those made from graphite particles and nanoparticles. However, for some practical applications, the use of noble metal electrodes has an edge over the other electrode systems. Moreover, a long biosensor lifespan is desirable to ensure the versatility of the end applications. In this work, a PQQ-GDH based biosensor was developed using chromium hexacyanoferrate (CrHCF) as a mediator to transfer electrons between the enzyme system and a Pt electrode. This biosensor was found to be very durable. Its proposed detection mechanism is shown in Scheme 1.

In the development of the glucose biosensor described herein, CrHCF was electrochemically deposited on a Pt electrode surface. Efforts were made to validate the presence of CrHCF on the Pt surface using a variety of technique such as the analysis of cyclic voltammograms (CVs), scanning electron microscopy (SEM), Fourier Transform infrared (FTIR) spectroscopy, and X-ray photoelectron spectroscopy (XPS). To become a functional glucose biosensor, PQQ-GDH was immobilized within the network of a cation exchange resin layer, Nafion, placed on the CrHCF/Pt electrode. Electrochemical Impedance spectroscopy (EIS) was used to substantiate the successful immobilization of the enzymes onto the electrode surface. As indicated in the glucose detection mechanism, the presence of Fe in the Fe^III^ state on the electrode surface is essential during the measurement of glucose. A potential treatment technique is applied to ensure that this prerequisite is met. A similar technique called the potential booster is used prior to the glucose measurement to remain the amplitude of the current response. The roles of the electric potential treatment and potential booster in the glucose detection mechanism were examined by X-ray photoelectron spectroscopy (XPS). The life-time of the biosensor was also evaluated.

## 2. Experimental Section

### 2.1. Materials

PQQ-GDH (EC 1.1.99.17 from Acinetobacter, grade III, 1,070 units/mg) is soluble and was obtained from Toyobo (Japan). d-(+)-Glucose, potassium ferro- and ferricyanide, potassium phosphate, and potassium chloride (KCl) were purchased from Riedel-deHaen (Germany). Chromium nitrate Cr(NO_3_)_3_ was obtained from Sigma-Aldrich (Germany). Nafion dispersion, 20–22 wt.% solution was purchased from DuPont (USA). The solution used in the preparation of the biosensors and in the measurements of response currents was phosphate buffer solution, PBS, (0.1 M, pH 7.0) containing K_2_HPO_4_ and KH_2_PO_4_. Glucose stock solutions were equilibrated for at least 24 hours prior to use.

### 2.2. Apparatus

The primary apparatus used in this work was a set of three electrodes, comprising an Ag/AgCl (3 M KCl) reference electrode, a Pt wire counter electrode, and a 2 mm-diameter Pt disc working electrode (CH Instruments Inc., USA). All of the potentials that were applied to the working electrode in the electrochemical experiments were with respect to the Ag/AgCl reference electrode. Before the working electrode was modified, it was pre-polished with a nylon pad and a 1,200 grit polish pad. Electrochemical experiments were performed with a Potentiostat/Galvanostat Autolab PGSTAT-10 (Eco chemie, Utrecht, Holland,) which was connected to a computer that ran GPES (General Purpose Electrochemical System) software.

### 2.3. Preparation of Chromium Hexacyanoferrate Modified Platinum Discs

The presence of CrHCF on the Pt electrode surface was confirmed using four disc-shaped Pt foils, each with a diameter of 15 mm. Prior to use, these foils were thoroughly cleaned by immersion in a solution that contained 30% H_2_O_2_ and 70% H_2_SO_4_ (v/v) for 12 hours. Three compounds, K_3_Fe(CN)_6_, Cr(NO_3_)_3_, and a mixture of the two, were individually prepared in 0.1 M KCl electrolyte and then were deposited electrically onto the three Pt foils, one compound per foil, by cycling the potential between −0.2 and +1.0 V at a scan rate of 25 mV s^−1^ for 250 cycles. These three compound-deposited foils and one bare Pt foil were potentio-dynamically swept between −0.2 and +1.0 V at a scan rate of 25 mV s^−1^ in a fresh 0.1 M KCl solution to obtain the voltammograms. The surface morphology of these four Pt foils was examined by SEM imaging, using a Quanta 400 FEG (FEI Company, Oregon, USA). A Fourier transform infrared reflection spectrometer, FTIR-4200 ATR PRO410-S (JASCO, Japan), was used to determine which foil contained CrHCF, by identifying the typical infrared absorption wavelength of CrHCF to be determined.

### 2.4. Fabrication of Chromium Hexacyanoferrate Modified Glucose Biosensor

The CrHCF-modified glucose biosensor was prepared using the method of Lin *et al.* [20], with the exception that the electrode was Pt rather than glassy carbon. The Pt working electrode was prepared by the cycling of potentials between −0.2 and +1.0 V for 30 cycles at a scan rate of 25 mV s^−1^ in 0.1 M KCl solution (pH 3) that contained 10 mM Cr(NO_3_)_3_ and 5 mM K_3_Fe(CN)_6_. This procedure formed a compound and deposited it on the surface of the Pt disc. According to Lin *et al.*, the deposited compound consists of mixed-valence clusters, each of which comprises of a few metal centers that are linked by a bridging ligand [20]. To provide a platform to immobilize the PQQ-GDH enzyme system a Nafion layer was then coated onto the CrHCF modified electrode as by adding a controlled volume of 0.25% (v/v) Nafion solution (typical volume 1 μL) onto the electrode surface and allowing it to dry in the ambient atmosphere. The Nafion-coated electrode absorbed the PQQ-GDH in the enzyme solution (3 mg mL^−1^) that was prepared in the PBS at a constant potential of +0.9 V for 15 minutes. Another Nafion layer served as a second matrix to immobilize the PQQ-GDH. The Nafion solution used herein was 0.5% (v/v) rather than 0.25%. Notably, the electrode underwent a potential pre-treatment procedure before the fabrication process was completed. The current response was empirically optimized by applying to the electrode in the PBS a constant voltage of +1.0 V three times, each for 30 minutes, on three consecutive days. The final CrHCF-modified PQQ-GDH-based glucose biosensor, CrHCF/PQQ-GDH, was stored in the PBS at room temperature when it was not being used.

### 2.5. Electrochemical Measurements

In this work, electrochemical measurements of glucose were made to determine the linear range of detection and the stability of the developed CrHCF/PQQ-GDH glucose biosensor. The measurements were made at an operating voltage of +0.4 V in 0.1 M PBS (pH 7.0) at room temperature. A constant voltage of +1.0 V for 60 s was applied to the bioelectrode before the amperometric measurements were made whenever the glucose biosensor remained unused overnight.

## 3. Results and Discussion

### 3.1. Confirmation of Presence of Chromium Hexacyanoferrate on the Pt Electrode

**Figure 1.**
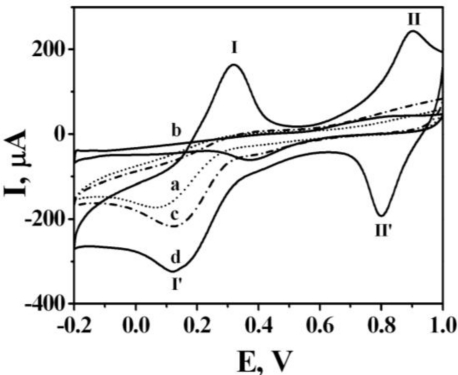
Typical cyclic voltammograms of compounds electrochemically deposited on surface of Pt foils; **(a)** bare Pt foil, **(b)** Cr(NO_3_)_3_, **(c)** K_3_Fe(CN)_6_, and **(d)** mix of Cr(NO_3_)_3_ and K_3_Fe(CN)_6_ in 0.1 M KCl electrolyte, pH = 3. Sweep cycles, 250 cycles.

**Figure 2.**
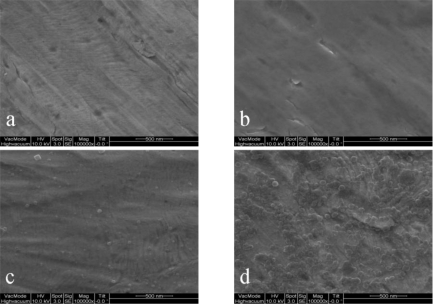
Scanning electron micrographs of compounds deposited on Pt foil surface; **(a)** bare, **(b)** electrochemically deposited with Cr(NO_3_)_3_, **(c)** K_3_Fe(CN)_6_ and **(d)** mixture of Cr(NO_3_)_3_ and K_3_Fe(CN)_6_ in 0.1 M KCl electrolyte, pH = 3.

**Figure 3.**
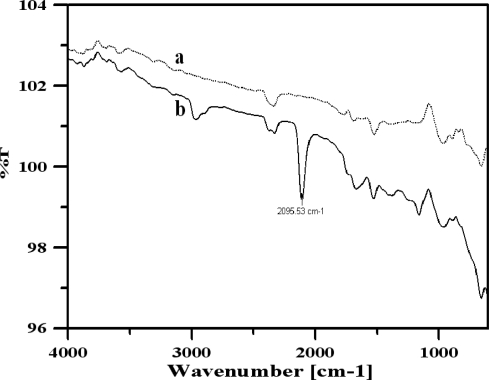
Fourier transform infrared spectra of Pt electrode surface: **(a)** bare, and **(b)** coated with chromium hexacyanoferrate. Other conditions are as given in Figure 1.

Figure 1 presents four typical CVs. Three of them, 1(b-d), were produced from Pt foils that were prepared in solutions of Cr(NO_3_)_3_, K_3_Fe(CN)_6_, and a mixture of Cr(NO_3_)_3_ and K_3_Fe(CN)_6_, respectively. The CV (a) in Figure 1 from the bare Pt foil served as a comparison, suggesting the presence of compounds on the other Pt foils. The corresponding reductive peaks of the CVs (b) and (c) were located at +0.383 V and +0.134 V, respectively. Unlike the CVs (b) and (c), the CV (d) has reductive peaks and oxidative peaks. Moreover, its redox peak pairs are at I, I’ (+0.315 V, +0.120 V) and II, II’ (+0.894 V, +0.801 V) which positions differ from those of the reductive peaks of CVs (b) and (c), suggesting that the deposited compound on foil d differs from those on foils (b) and (c). The SEM images of the four foils in Figure 2 were ordered in the same way as the CVs in Figure 1. In photograph 2(b), the surface seems to be covered by a smooth membrane. In photograph 2(c), small grains are formed on the foil surface in a dispersed fashion. The large and clustered grains at the surface in photograph 2(d) differ significantly from those observed in photographs 2(b) and 2(c). To confirm that the grains in photograph 2(d) are CrHCF, the bare Pt foil and the foil that was modified using the mixed solution of Cr(NO_3_)_3_ and K_3_Fe(CN)_6_ were further examined using an FTIR spectrometer. Figure 3 shows the results; curves 3(a) and 3(b) refer to the IR adsorption spectra of the bare Pt foil and the mixed solution-modified Pt foil, respectively. The major difference between the two IR spectra is that the band at 2,093 cm^−1^ that appears in curve (b) but not in curve (a), and attributed to the C≡N moiety [21,22]. XPS, rather than FTIR, was used to verify the presence of Fe ions in the compound, because the XPS spectra can be applied to identify the typical binding energies of Fe(II, III). Additionally, the evidence for conversion of Fe(II) to Fe(III) by potential treatment and potential boosting is strong.

### 3.2. Electrochemical Impedance Spectroscopy

**Figure 4.**
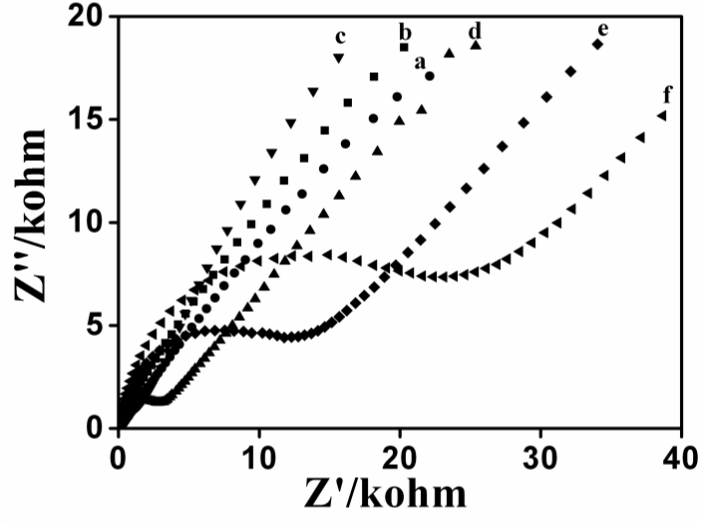
Electrochemical impedance spectra of **(a)** bare Pt, **(b)** CrHCF/Pt, **(c)** Nafion/CrHCF/Pt, **(d)** PQQ-GDH/CrHCF/Pt, **(e)** PQQ-GDH/Nafion/CrHCF/Pt and **(f)** Nafion/PQQ-GDH/Nafion/CrHCF/Pt electrodes in the solution containing K_3_Fe(CN)_6_ and K_4_Fe(CN)_6_ (1 mM each) and 0.1M KCl.

Electrochemical impedance spectroscopy was performed to characterize the interfacial properties of the modified electrodes and to corroborate the successful immobilization of the PQQ-GDH onto the modified surface. Figure 4 shows the impedance spectra recorded with various electrodes: (Pt, CrHCF/Pt, PQQ-GDH/CrHCF/Pt, Nafion/CrHCF/Pt, PQQ-GDH/Nafion/CrHCF/Pt and Nafion/PQQ-GDH/Nafion/CrHCF/Pt) in 0.1 M KCl solution containing Fe^II^/Fe^III^ ions.

One can observe distinct differences in the impedance spectra, but all the spectra follow the theoretical shapes and include a semicircular portion at higher frequencies that corresponds to the charge transfer limited process (R_ct_), followed by a linear part at lower frequency, due to a diffusionally limited electron transfer process. The charge transfer resistance controls the electron-transfer kinetics of the redox probe at the electrode/electrolyte interface.

Both bare Pt and CrHCF/Pt electrodes 4(a,b) exhibited virtually straight lines, a typical characteristic of a diffusion limiting step of the electrochemical process, the CrHCF/Pt electrode covered with Nafion layer 4(c) also showed an almost straight line. After modification of the Nafion/CrHCF/Pt with PQQ-GDH 4(e), R_ct_ reached a value of about 17.32 kΩ. This R_ct_ increase to 17.32 kΩ emphasizes the bio-layer formation on the Nafion/CrHCF/Pt electrode. The enzymes are expected to block the electron transfer of the redox probe at the modified electrode surface. An alternative electrode is fabricated by immobilization of PQQ-GDH enzymes directly onto the surface of CrHCF/Pt electrodes (without using the Nafion layer) and the impedance response was measured 4(d) and compared with PQQ-GDH/Nafion/CrHCF/Pt electrode 4(e). A relatively low R_ct_ (2.12 kΩ) was observed at PQQ-GDH/CrHCF/Pt electrode 4(d). It is clear that the immobilization of PQQ-GDH on Nafion/CrHCF/Pt rather than at CrHCF/Pt electrode leads to a stable assembly. An R_ct_ of about 29.83 kΩ was observed for the final biosensor electrode, Nafion/PQQ-GDH/Nafion/CrHCF/Pt 4(f). These results demonstrate the successful immobilization of PQQ-GDH enzymes onto the electrode assembly.

### 3.3. Effects of Potential Pre-Treatment and Potential Boosting

The stability and the current of the CrHCF/PQQ-GDH biosensor are improved considerably when the electrode was potentially pre-treated before each of the measurement. The experiments reveal that the glucose current responses of as fabricated CrHCF/PQQ-GDH biosensor were rather low when the biosensor did not undergo any potential treatment.

Interestingly, when the biosensor was potentially treated, the measured glucose current responses were amplified. Also, it was found that when the biosensor was not used and was stored in the PBS at room temperature overnight or longer, the responses were weakened markedly. In that case, the biosensor required a potentially pre-treated and this was ascertained by application of +1.0 V for 60 s in the PBS before the measurements. A single potential boost caused the amplitudes of the glucose current response to return to the normal range, where it remained throughout the following measurements.

**Figure 5.**
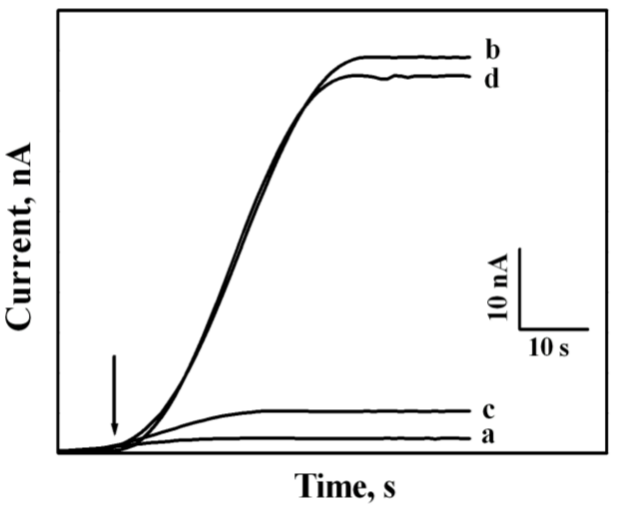
Amperometric responses of CrHCF/PQQ-GDH glucose biosensor in scenarios when it was **(a)** newly completed, **(b)** potentially treated, **(c)** unused overnight, and **(d)** potentially boosted in detecting 5 mM glucose in 0.1 M phosphate buffer (pH 7). Arrow indicates time of spiking with 5 mM glucose.

Figure 5 shows the current responses, measured from 5 mM glucose at an operating potential of +0.4V using the CrHCF/PQQ-GDH biosensor. The labels a, b, c, and d in Figure 5 correspond to the responses detected by the biosensor in the scenarios that the biosensor was newly completed, potentially treated, long-unused, and potentially boosted, respectively. Such a glucose response pattern obtained following potential treatment and potential boosting remained consistent throughout this work.

### 3.4. XPS Spectral Analysis

**Figure 6.**
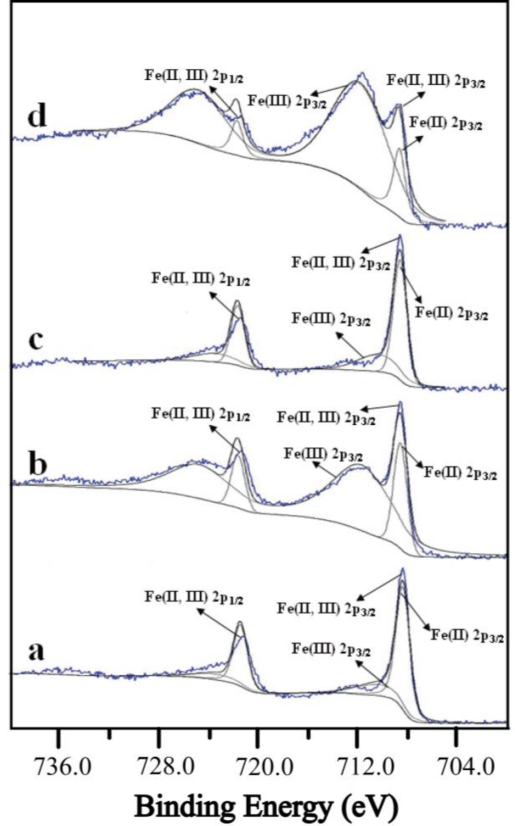
XPS analysis of CrHCF-modified Pt discs when they were: **(a)** newly modified, **(b)** potentially treated, **(c)** unused overnight, and **(d)** potentially boosted.

Four CrHCF-deposited Pt discs were prepared for XPS analysis to elucidate the aforementioned glucose response pattern. Such a pattern is believed to be associated with the initial form of the mediator, either KCr^III^Fe^II^(CN)_6_ or Cr^III^Fe^III^(CN)_6_, existing in the proposed glucose detection mechanism. For identification, each of the CrHCF-coated discs was processed to represent a simple form of the biosensor in the four scenarios described above. Each was scanned using an XPS instrument (PHI Quantera SXM/Auger: AES 650). Since the major concern was the iron cations in the CrHCF that was deposited on the surface of the Pt discs, the XPS analysis aimed on Fe 2p_3/2_ envelope peaks and their curve-fitted Fe(II) and Fe(III) peaks, as shown in Figure 6.

Although the presence of Cr^III^Fe^III^(CN)_6_ is required, KCr^III^Fe^II^(CN)_6_ was present on the surface of the Pt electrode at the formation of the CrHCF/PQQ-GDH biosensor, as shown by the XPS spectra of the Pt disc onto which CrHCF had just been deposited. Its Fe 2p_3/2_ envelope and fitted peaks are shown in Figure 6(a), in which only a binding energy peak at 708.3 eV is evident. However, Grosvenor *et al.* found an Fe(II) 2p_3/2_ binding energy peak of K_4_Fe^II^(CN)_6_ at 708.4 eV and Fe(III) 2p_3/2_ peaks of FeF_3_, FeCl_3_ and FeBr_3_ from 710.3 to 715.1 eV [23]. The binding energy peak of 708.3 eV implies that the primary CrHCF compound on the biosensor in this stage was KCr^III^Fe^II^(CN)_6_, which inhibits the electron relay from the site of GDH-PQQ to the Pt electrode, resulting in a very weak current response. The Fe 2p_3/2_ XPS spectra of the CrHCF-deposited Pt disc to which was applied a potential of +1.0 V for 90 minutes verified the increase in the current response of the biosensor upon potential treatment, as presented in Figure 6(b).

The fitted peaks of Fe(II) and Fe(III) at 708.6 and 712.0 eV, respectively, indicate that a potential of +1.0 V oxidized KCr^III^Fe^II^(CN)_6_ to Cr^III^Fe^III^(CN)_6_. The approach was based on the work of Dostal who found that the formal potential of a potassium ion is between +0.7 and +0.76 V [24]. Hence, the potential +1.0 V supports the equation:

$${\mathrm{KCr}}^{\mathrm{III}}{\mathrm{Fe}}^{\mathrm{II}}{\left(\mathrm{CN}\right)}_{6}\overset{+1.0V}{\to }K^{+}+{\mathrm{Cr}}^{\mathrm{III}}{\mathrm{Fe}}^{\mathrm{III}}{\left(\mathrm{CN}\right)}_{6}+e^{-}$$

The previous discussion proves that the potential treatment procedure made the mediator in the form of Cr^III^Fe^III^(CN)_6_ before the detection. This ensures that the proposed mechanism is accountable. The electron that is generated by the reaction of glucose and GDH is relayed by PQQ and accepted by Cr^III^Fe^III^(CN)_6_ to yield Cr^III^Fe^II^(CN)_6_, which then returns to the form Cr^III^Fe^III^(CN)_6_ with the release of the electron to the electrode. Thus, the biosensor measured the glucose response currents. After the biosensor had been idle and stored in the PBS overnight, its response current in the detection of glucose was substantially reduced. Such a biosensor is believed to undergo the reduction of Cr^III^Fe^III^(CN)_6_ to KCr^III^Fe^II^(CN)_6_ while idle, limiting the electron transfer in the detection of glucose. Figure 6(c) shows the XPS spectra of the third CrHCF Pt disc following potential treatment and left unused overnight in the PBS. This figure shows the reduction; the fitted peaks of Fe(II) and Fe(III) are at 708.6 and 710.7 eV, respectively, with the fitted peak of Fe(II) being dominant. This result suggests that Cr^III^Fe^III^(CN)_6_ was reduced to KCr^III^Fe^II^(CN)_6_ spontaneously when the biosensor was left idle for an extended period. Such a reduction was minimal in the very first hour of inactivity, as revealed by the fact that, in the corresponding experiments, the drop in the current response was barely noticeable. The decline caused by the reduction can be recovered by the potential boosting technique described above. As in the above verification studies, this potential boost effect is identified by analysis of the XPS spectra of the fourth CrHCF Pt disc that underwent potential treatment, left idle overnight, and underwent potential boosting, as shown in Figure 6(d). In this figure, the fitted peaks of Fe(II) and Fe(III) are at 708.7 and 712.1 eV, respectively. The dominant peak is Fe(III) in this stage implying that the majority of the mediator is of the form Cr^III^Fe^III^(CN)_6_, facilitating the detection of glucose, as described above.

### 3.5. Effect of Glucose Concentration

**Figure 7.**
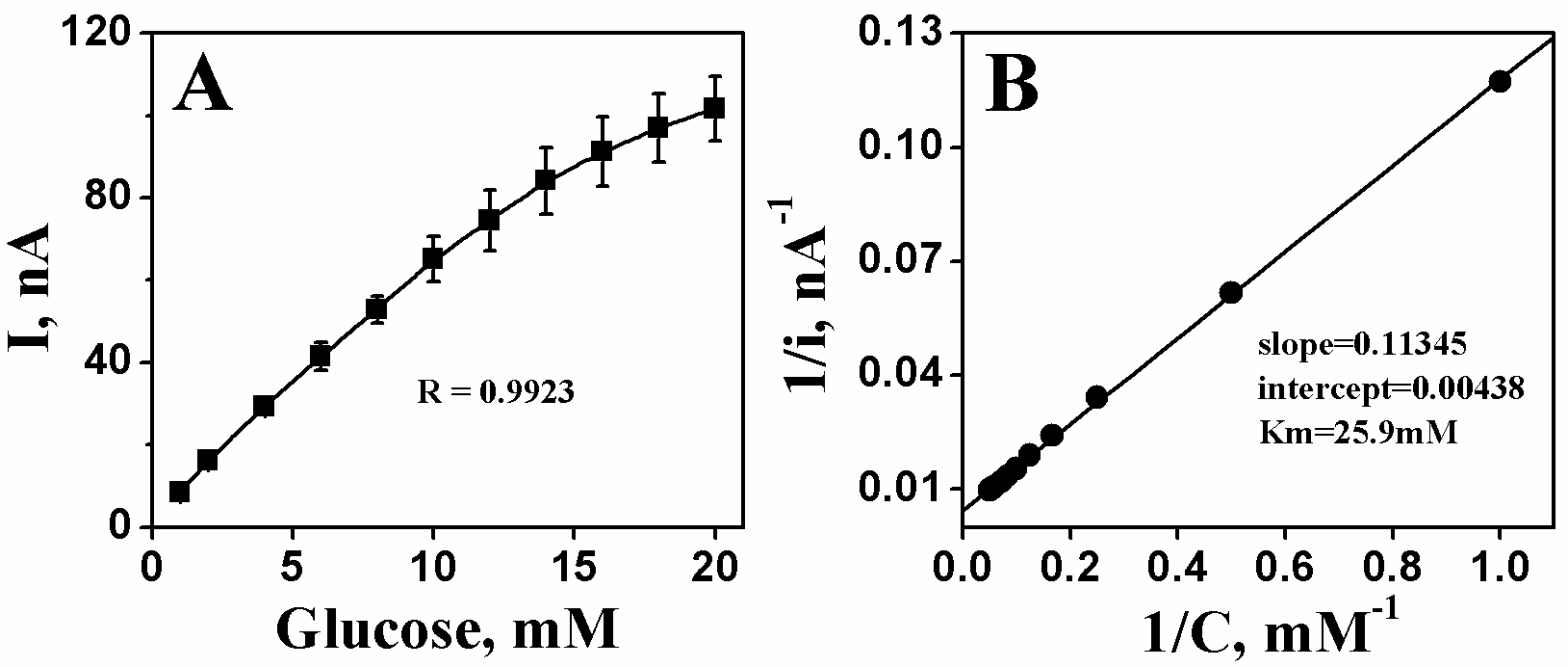
**(A)** Typical calibration curve of CrHCF/PQQ-GDH biosensor in determination of concentration of glucose. Conditions are as given in Figure 5. **(B)** A Lineweaver-Burke plot derived from the calibration curve in (A).

Figure 7 shows the amperometric current response with respect to the glucose concentration of the developed CrHCF/PQQ-GDH glucose biosensor. In this experiment, the biosensor was tested by applying an operating potential of +0.4 V at room temperature. The tested glucose concentrations ranged from 1 to 20 mM; the first two injections were of 1 mM and the rest were of 2 mM. This result reveals that the linear range of the calibration plot was at a level of 20 mM (correlation coefficient R = 0.9923) with a sensitivity of 199.94 nA/mM per cm^2^. The catalytic characteristic change of the PQQ-GDH enzyme system after immobilization was evaluated by calculating an apparent Michaelis-Menten constant, *K_m_*, under steady-state conditions. Unlike from the *K_m_* value specified by the supplier, 4.8 mM, the *K_m_* value of the developed glucose biosensor, calculated from a Lineweaver-Burke plot, was 25.9 mM. The difference indicates that the characteristic of the PQQ-GDH was altered due to the immobilization procedure especially with the addition of the two layers of Nafion. The support of these two layers not only assured the stability of the PQQ-GDH but also likely caused the developed glucose biosensor to operate in a diffusion mode.

### 3.6. Oxygen Interference

**Figure 8.**
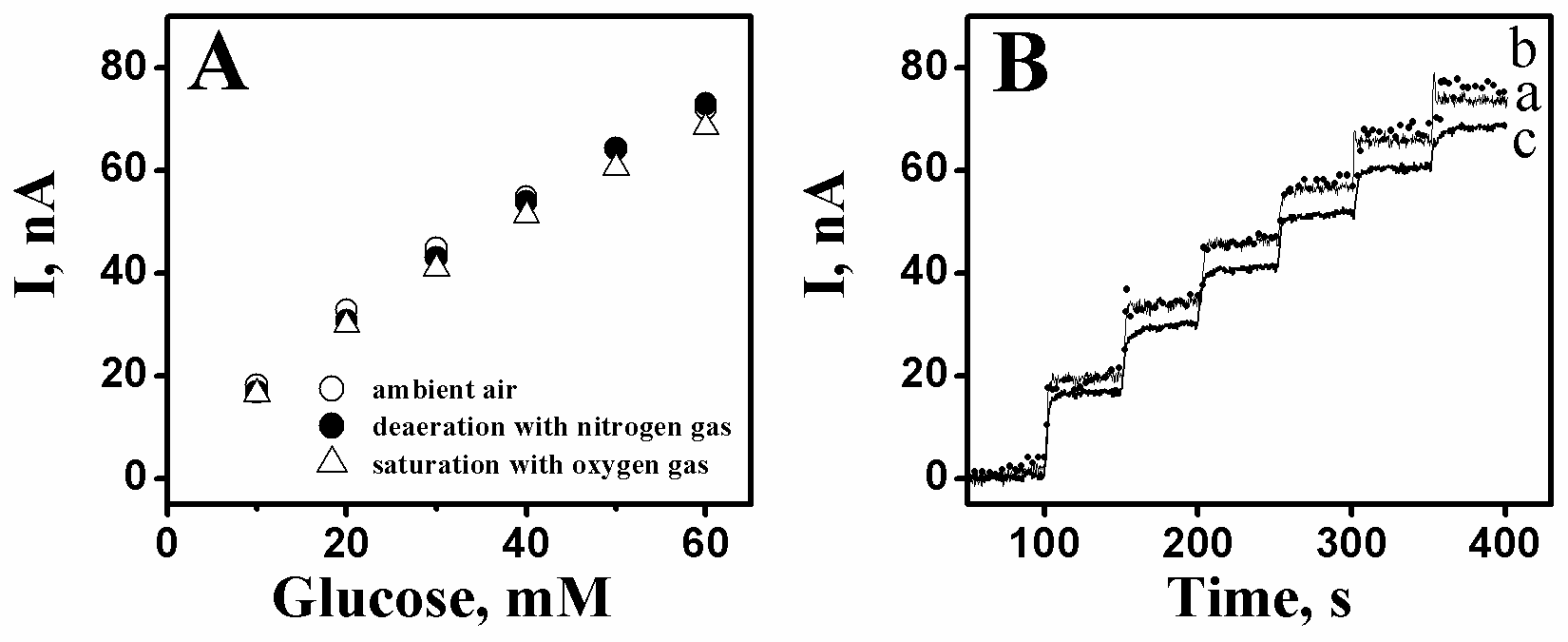
**(A)** Amperometric responses of CrHCF/PQQ-GDH glucose biosensor in determination of concentration of glucose in 0.1 M phosphate buffer (pH 7) at the conditions of ambient air (○), deaeration with nitrogen gas (•), and saturation with oxygen gas (Δ). **(B)** The glucose responses of the biosensor along the time when the glucose buffers at the conditions of (a) ambient air, (b) deaeration with nitrogen gas, and (c) saturation with oxygen gas. Stirring speed: 300 rpm; Operating temperature: 25 °C; Applied potential: +1.0 V.

The question if oxygen serves as a mediator accepting electrons from the enzyme, producing hydrogen peroxide, and then oxidation of hydrogen peroxide taking place on CrHCF is raised. To address this question, an amperometric response study of the CrHCF/PQQ-GDH glucose biosensor in the determination of concentration of glucose in 0.1 M PBS (pH 7) under the conditions of: (1) ambient air, (2) deaeration with nitrogen gas, and (3) saturation with oxygen gas was performed. In the PBS containing glucose, both the procedures of degasification of nitrogen and saturation of oxygen lasted for 20 minutes and the potential booster technique was applied before the measurements. The study results showed that the glucose responses measured in these three conditions were almost indistinguishable, as shown in Figure 8. This proves that when the CrHCF/PQQ-GDH biosensor detects glucose, oxygen does not play the role of electron acceptor. As a result, no hydrogen peroxide is produced in this detection mechanism.

### 3.7. Stability Performance

**Figure 9.**
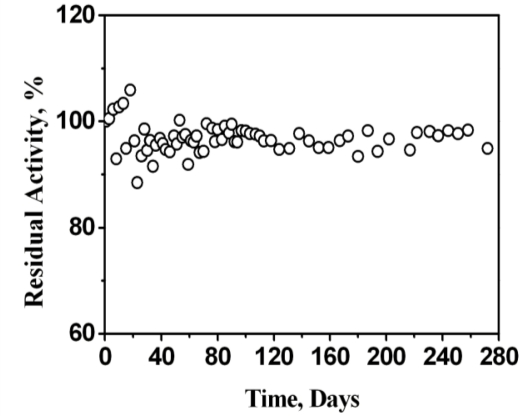
Stability performance of CrHCF/Pt electrode. Conditions are as given in Figure 5.

To the best of the authors’ knowledge, most mediators that are used in conjunction with the enzyme system PQQ-GDH have difficulty retaining stable response currents over time. In contrast, the stability of the CrHCF/PQQ-GDH glucose biosensor is excellent, provided that potential treatment and potential boosting procedures are applied. Since the determination of stability was a long-term assessment, when the biosensor was not in use, it was stored in the PBS at room temperature. When it was used in the test, the response current was measured upon the injection of 5 mM glucose into the PBS at +0.4 V at room temperature. In each test, three glucose responses were obtained and averaged to yield the test data. Notably, no more than one test was conducted daily. The time intervals between consecutive tests were random. Sixty-eight tests were performed over 272 days. All test data were normalized to the data obtained on the very first day when the biosensor had just been fabricated. Figure 9 plots residual activity (as normalized ratios) as a function of the number of days. Throughout the evaluation of stability performance, the residual activity of the biosensor did not fall below 88%. Given such performance, the CrHCF/PQQ-GDH glucose biosensor is regarded as very stable. The performance (in terms of lifespan and sensitivity) of CrHCF/PQQ-GDH as glucose sensor is compared with earlier reported PQQ-GDH based glucose sensors and is presented in Table 1.

## 4. Conclusions

A glucose biosensor that is based on the PQQ-GDH enzyme system, modified with the mediator CrHCF on a Pt electrode, was developed. The presence of CrHCF on the Pt electrode surface was confirmed. When CrHCF is newly synthesized on the Pt electrode surface, the predominance of Fe(II) in CrHCF was verified, which restrains the proposed glucose detection mechanism that requires Fe(III). This problem was overcome by using a potential treatment during the fabrication of the biosensor. The potential treatment causes the conversion of Fe(II) to Fe(III) in the CrHCF. A similar potential boost technique applied before the amperometric measurements of glucose were made, extended the functional lifetime of the biosensor. In this work, the conversion of CrHCF from reduction form to oxidation form so that it becomes a useful mediator in conjunction with PQQ-GDH demonstrated the practical use of the potential treatment techniques. Analogous studies, such as research on mediator redox forms that are needed in applications, convertible mediators, potential voltages that are essential, optimal time length of potential treatment, *etc*, can be further extended.

## Figures and Tables

**Table 1.** Comparison of the performance of different PQQ-GDH based glucose biosensors.

**Electrode material**	**Mediator**	**Applied potential**	**Lifespan (50%)**	**Current density** (mA × mM^−1^ × cm^−2^)	**Ref.**

Graphite	Os-complex	+0.2 V	>12 hours	5.9	[4]
Carbon	4-ferrocenylphenol	+0.3 V	—	2	[1]
Carbon	4-ferrocenylphenol	+0.4 V	>21 days	3	[10]
Glassy Carbon	N-methylphenazonium	+0.3 V	—	6 × 10^−5^	[9]
Gold	Au nanoparticles	+0.7 V	—	—	[14]
Carbon paste	Ferricyanide	+0.5 V	>30 days	31	[12]
Platinum	Chromium hexacyanoferrate	+0.4 V	>270 days	2 × 10^−4^	This work
